# Case Reports, Case Series – From Clinical Practice to Evidence-Based Medicine in Graduate Medical Education

**DOI:** 10.7759/cureus.1546

**Published:** 2017-08-07

**Authors:** Jerry W Sayre, Hale Z Toklu, Fan Ye, Joseph Mazza, Steven Yale

**Affiliations:** 1 Family Medicine, North Florida Regional Medical Center; 2 Graduate Medical Education, North Florida Regional Medical Center; 3 Department of Clinical Research, Marshfield Clinic Research Foundation; 4 Internal Medicine, University of Central Florida College of Medicine

**Keywords:** clinical, case reports, case studies, evidence-based medicine, graduate medical education, resident training, residency, clinical skills, publishing, clinical practice

## Abstract

Case reports and case series or case study research are descriptive studies that are prepared for illustrating novel, unusual, or atypical features identified in patients in medical practice, and they potentially generate new research questions. They are empirical inquiries or investigations of a patient or a group of patients in a natural, real-world clinical setting. Case study research is a method that focuses on the contextual analysis of a number of events or conditions and their relationships. There is disagreement among physicians on the value of case studies in the medical literature, particularly for educators focused on teaching evidence-based medicine (EBM) for student learners in graduate medical education. Despite their limitations, case study research is a beneficial tool and learning experience in graduate medical education and among novice researchers. The preparation and presentation of case studies can help students and graduate medical education programs evaluate and apply the six American College of Graduate Medical Education (ACGME) competencies in the areas of medical knowledge, patient care, practice-based learning, professionalism, systems-based practice, and communication. A goal in graduate medical education should be to assist residents to expand their critical thinking, problem-solving, and decision-making skills. These attributes are required in the teaching and practice of EBM. In this aspect, case studies provide a platform for developing clinical skills and problem-based learning methods. Hence, graduate medical education programs should encourage, assist, and support residents in the publication of clinical case studies; and clinical teachers should encourage graduate students to publish case reports during their graduate medical education.

## Editorial

Introduction

Case reports and case series or case study research* *are descriptive studies to present patients in their natural clinical setting. Case reports, which generally consist of three or fewer patients, are prepared to illustrate features in the practice of medicine and potentially create new research questions that may contribute to the acquisition of additional knowledge in the literature. Case studies involve multiple patients; they are a qualitative research method and include in-depth analyses or experiential inquiries of a person or group in their real-world setting. Case study research focuses on the contextual analysis of several events or conditions and their relationships [1]. In addition to their teaching value for students and graduate medical education programs, case reports provide a starting point for novice investigators, which may prepare and encourage them to seek more contextual writing experiences for future research investigation. It may also provide senior physicians with clues about emerging epidemics or a recognition of previously unrecognized syndromes. Limitations primarily involve the lack of generalizability and implications in clinical practice, which are factors extraneous to the learning model (Table 1).

**Table 1:** Advantages and disadvantages of case reports and case studies

Advantages	Disadvantages
One case to initiate a signal (case report)	No control (uncontrolled)
Provide stronger evidence with multiple cases (cases series)	Difficult to compare different cases
Observational	Cases may not be generalizable
Educational	Selection bias
Easy to do (fast and no financial support needed)	Unknown future outcome/follow-up
Identify rare manifestations of a disease or drug	

**Figure 1:**
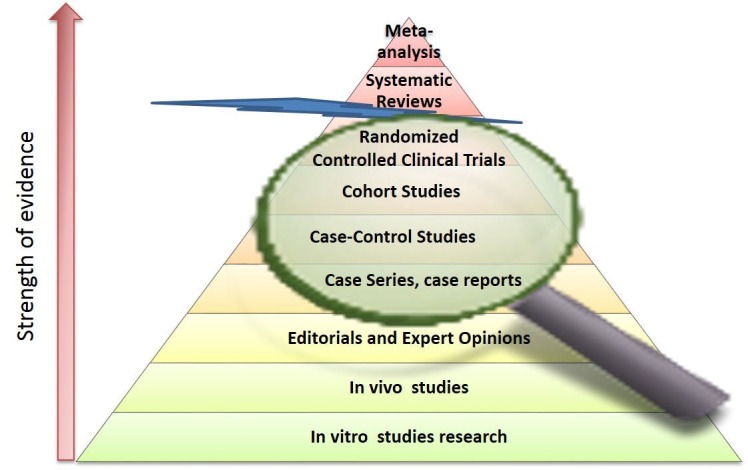
Revised pyramid for study designs and their place in evidence-based medicine Modified from Murad et al. [2]

There is disagreement among physicians on the value of case reports in the medical literature and in evidence-based medicine (EBM) [2]. EBM aims to optimize decision-making by using evidence from well-conducted research. Therefore, not all data has the same value as the evidence. The pyramid (Figure 1) classifies publications based on their study outlines and according to the power of evidence they provide [2-3]. In the classical pyramid represented below, systematic reviews and a meta-analysis are expected to provide the strongest evidence. However, a recent modification of the pyramid was suggested by Murad et al. [2]: the meta-analysis and systematic reviews are removed from the pyramid and are suggested to be a lens through which evidence is viewed (Figure 1). 

Because case reports do not rank highly in the hierarchy of evidence and are not frequently cited, as they describe the clinical circumstances of single patients, they are seldom published by high-impact medical journals. However, case reports are proposed to have significant educational value because they advance medical knowledge and constitute evidence for EBM. In addition, well-developed publication resources can be difficult to find, especially for medical residents; those that do exist vary in quality and may not be suitable for the aim and scope of the journals. Over the last several years, a number (approximately 160) of new peer-reviewed journals that focus on publishing case reports have emerged. These are mostly open-access journals with considerably high acceptance rates [4]. Packer et al. reported a 6% publication rate for case reports [5]; however, they did not disclose the number of papers submitted but rejected and neither did they state whether any of the reported cases were submitted to open-access journals.

The development of open-access journals has created a new venue for students and faculty to publish. In contrast to subscription-based and peer-reviewed e-journals, many of these new case report journals are not adequately reviewed and, instead, have a questionably high acceptance rate [4]. There, however, remains the issue of the fee-based publication of case reports in open-access journals without proper peer reviews, which increases the burden of scientific literature. Trainees should be made aware of the potential for academic dilution, particularly with some open-access publishers. While case reports with high-quality peer reviews are associated with a relatively low acceptance rate, this rigorous process introduces trainees to the experience and expectations of peer reviews and addresses other issues or flaws not considered prior to submission. We believe that these are important skills that should be emphasized and experienced during training, and authors should seek these journals for the submission of their manuscripts.

Importance of Case Reports and Case Series in Graduate Medical Education

**Figure 2:**
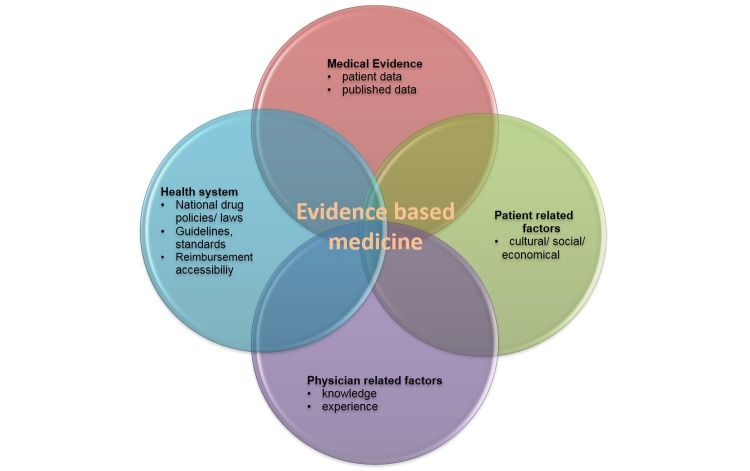
Factors involved in decision making with the context of evidence-based medicine Modified from Toklu et al. 2015 [3]

The Accreditation Council for Graduate Medical Education (ACGME) has challenged faculties to adapt teaching methodologies to accommodate the different learning modalities of the next generation of physicians. As evidenced by its implementation by ACGME, competency-based medical education is rapidly gaining international acceptance, moving from classic didactic lectures to self-directed learning opportunities with experiential learning aids in the development of critical cognitive and scholarly skills. As graduate medical educators, we are in agreement with Packer et al. about the value of the educational benefits resulting from student-generated case reports [5]. Case study assignments help residents develop a variety of key skills, as previously described. EBM is an eventual decision-making process for executing the most appropriate treatment approach by using the tools that are compatible with the national health policy, medical evidence, and the personal factors of physician and patient (Figure 2). The practice of identifying and developing a case study creates a learning opportunity for listening skills and appreciation for the patient’s narrative as well as for developing critical learning and thinking skills that are directly applicable to the practice of EBM. This critically important process simultaneously enhances both the medical and the humanistic importance of physician-patient interaction. In addition, case-based learning is an active learner-centered approach for medical students and residents. It serves as a curricular context, which can promote the retention of information and evidence-based thinking.

Conclusion

The value of case studies in the medical literature is controversial among physicians. Despite their limitations, clinical case reports and case series are beneficial tools in graduate medical education. The preparation and presentation of case studies can help students and residents acquire and apply clinical competencies in the areas of medical knowledge, practice-based learning, systems-based practice, professionalism, and communication. In this aspect, case studies provide a tool for developing clinical skills through problem-based learning methods. As a result, journals should encourage the publication of clinical case studies from graduate medical education programs through a commonly applied peer-review process, and clinical teachers should promote medical residents to publish case reports during their graduate medical education.
